# Insulin resistance associates with hepatic lobular inflammation in subjects with obesity

**DOI:** 10.1530/EC-19-0366

**Published:** 2019-08-29

**Authors:** Frederique Van de Velde, Marlies Bekaert, Anja Geerts, Anne Hoorens, Arsène-Hélène Batens, Samyah Shadid, Margriet Ouwens, Yves Van Nieuwenhove, Bruno Lapauw

**Affiliations:** 1 Department of Endocrinology, Ghent University Hospital, Ghent, Belgium; 2 Department of Hepatology, Ghent University Hospital, Ghent, Belgium; 3 Department of Pathology, Ghent University Hospital, Ghent, Belgium; 4 Institute of Clinical Biochemistry and Pathobiochemistry, German Diabetes Center at the Heinrich-Heine-University Duesseldorf, Leibniz Center for Diabetes Research, Duesseldorf, Germany; 5 German Center for Diabetes Research (DZD), Munich-Neuherberg, Germany; 6 Department of Gastrointestinal Surgery, Ghent University Hospital, Ghent, Belgium

**Keywords:** NAFLD, insulin resistance, hepatic inflammation, obesity

## Abstract

**Purpose:**

Obese subjects with nonalcoholic fatty liver disease (NAFLD) are more prone to develop additional metabolic disturbances such as systemic insulin resistance (IR) and type 2 diabetes. NAFLD is defined by hepatic steatosis, lobular inflammation, ballooning and stage of fibrosis, but it is unclear if and which components could contribute to IR.

**Objective:**

To assess which histological components of NAFLD associate with IR in subjects with obesity, and if so, to what extent.

**Methods:**

This cross-sectional study included 78 obese subjects (mean age 46 ± 11 years; BMI 42.2 ± 4.7 kg/m^2^). Glucose levels were analysed by hexokinase method and insulin levels with electrochemiluminescence. Homeostasis model assessment-estimated insulin resistance (HOMA-IR) was calculated. Liver biopsies were evaluated for histological components of NAFLD.

**Results:**

A positive association between overall NAFLD Activity Score and HOMA-IR was found (*r*_s_ = 0.259, *P* = 0.022). As per individual components, lobular inflammation and fibrosis stage were positively associated with HOMA-IR, glucose and insulin levels (*P* < 0.05), and HOMA-IR was higher in patients with more inflammatory foci or higher stage of fibrosis. These findings were independent of age, BMI, triglyceride levels, diabetes status and sex (all *P* < 0.043). In a combined model, lobular inflammation, but not fibrosis, remained associated with HOMA-IR.

**Conclusion:**

In this group of obese subjects, a major contributing histological component of NAFLD to the relation between NAFLD severity and IR seems to be the grade of hepatic lobular inflammation. Although no causal relationship was assessed, preventing or mitigating this inflammatory response in obesity might be of importance in controlling obesity-related metabolic disturbances.

## Introduction

An important complication of obesity is the development of nonalcoholic fatty liver disease (NAFLD), which covers a broad histological spectrum from simple steatosis to nonalcoholic steatohepatitis (NASH), and can progress to fibrosis, cirrhosis and even hepatocellular carcinoma. Nonalcoholic fatty liver (NAFL) is diagnosed when steatosis is present in >5% of the hepatocytes with or without nonspecific mild lobular inflammation, while patients with NASH require the joint presence of steatosis along with ballooning and lobular inflammation (1, 2). To date, liver biopsy is the reference standard for diagnosis of NAFLD stage and quantification of histological components of NAFLD. Currently, two frequently used evaluation methods are NAFLD Activity Score (NAS) and Steatosis, Activity, and Fibrosis (SAF) score (3).

Importantly, patients with obesity who develop NAFLD are also more prone to develop other metabolic consequences of obesity such as insulin resistance (IR) and subsequently type 2 diabetes (T2D) (4). The pathogenesis of NAFLD is complex whereby the historical two-hit hypothesis is replaced by a multiple parallel hits hypothesis also considering the impact of an altered adipokine secretory pattern, inflammation, gut microbiota, nutritional factors, genetic and epigenetic factors, and IR on the development and progression of NAFLD (5, 6). Especially this interrelation between IR and NAFLD is intriguing, as IR can be modified, and may lead to T2D. Recent experimental work suggested that excess accumulation of diacylglycerol (DAG) in hepatocytes, rather than hepatic steatosis *per se* (intrahepatic triglyceride (TG) content), is the main molecular mechanism of development of IR (7, 8). Until now, however, few human studies investigated the association between IR and individual histological components of NAFLD (steatosis, lobular inflammation, ballooning and fibrosis) (9, 10, 11, 12, 13, 14, 15). Moreover, most of these studies were not primarily designed to investigate IR, included non-obese or paediatric subjects, and yielded contradictory overall results. As an increase in knowledge and understanding of the complex relation between IR and NAFLD could lead to possible new therapeutic pathways or targets, we addressed this association specifically in a high-risk, severely obese population with histological evaluation of NAFLD.

## Materials and methods

### Study design and subjects

In a cross-sectional study (registration number B67020084018), 71 men with obesity who were scheduled for gastric bypass surgery (GBS) were included. These men met the national reimbursement criteria for GBS since they had either a BMI >40 kg/m^2^ or a BMI >35 kg/m^2^ with at least one of the following co-morbidities – T2D, obstructive sleep apnoea, therapy-resistant arterial hypertension. Exclusion criteria were malignancies, drinking more than three units alcohol per day, known liver pathologies other than NAFLD, and a recent diagnosis of hypo- or hyperthyroidism or a recent change in their medication. In total 16 subjects were excluded from the analysis; due to use of GLP-1 analogue (*n* = 1) or insulin (*n* = 7), refusing liver biopsy (*n* = 1), no scored liver biopsy (*n* = 4), no blood samples available (*n* = 1), or no pre-surgical blood samples (*n* = 2). In addition, 24 subjects with obesity were recruited in another study (registration number B670201526667) which had the same inclusion and exclusion criteria. One patient refused a liver biopsy. These 23 subjects consisted of 8 women and 15 men. Four of them started a conservative weight loss programme, the other 19 subjects underwent GBS. In both studies, glucose-lowering medication (for example, two subjects were on sulphonylurea and one subject used a DPP4-inhibitor) was discontinued at least 24 h before surgery. None of the subjects used vitamin E supplements, thiazolidinediones or known steatogenic medication. During the pre-operative period, body weight was stable, and no subjects used a very low caloric diet. Thus, a total of 78 subjects with obesity who underwent liver biopsy were available for analysis. All participants gave written informed consent to participate in these studies, which were approved by the Ethical Review Board of the Ghent University Hospital and conducted according to the principles of the Declaration of Helsinki.

### Anthropometrics and general characteristics

For all subjects standing height was measured to the nearest 0.1 cm using a wall-mounted stadiometer. Body weight was measured to the nearest 0.1 kg on a calibrated scale in light indoor clothing without shoes. Subsequently, BMI was calculated. T2D was defined according to the ADA criteria (16). Medication use was retrieved from the patients’ records and double checked by questioning the patient.

### Biochemical measurements

Blood samples were collected after overnight fasting. All blood samples were centrifuged, serum was fractionated and stored at −80°C until further analysis.

Serum levels of fasting glucose were analysed by the hexokinase method (COBAS, Roche Diagnostics). Insulin levels were determined with electrochemiluminescence using the immunoanalyzer COBAS e411 (Roche Diagnostics). Homeostasis model assessment-estimated insulin resistance (HOMA-IR) was calculated using the following formula (17):


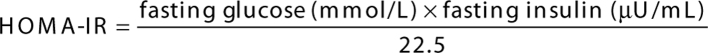


C-reactive protein (CRP), TG, cholesterol and high-density lipoproteins (HDL) were routinely determined using standard laboratory assays (COBAS 8000 modular analyser series, Roche Diagnostics, Mannheim, Germany). Low-density lipoproteins (LDL) was calculated using the Friedman formula: 


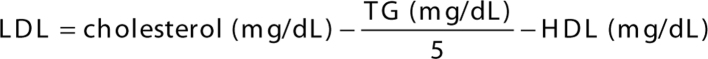


Non-esterified fatty acids (NEFAs) were determined with the standard enzymatic colorimetric method (P-modular; Roche Diagnostics).

### Hepatic histopathological analysis

Liver biopsies from the four patients following the conservative approach were taken, guided by ultrasonography using a biopsy gun with an 18-gauge needle (Bard Magnum, Tempe, AZ, USA) after local anaesthesia with 2% xylocaine. In the other participants, a liver biopsy was carried out at the end of the GBS procedure, which measured at least 5 × 5 mm and was taken from the lateral edge of the left liver lobe. All biopsies were immediately fixed in formalin (buffered 4% formaldehyde solution, Klinipath, Belgium) at room temperature for microscopic analysis. The formalin-fixed, paraffin-embedded tissue sections were stained with hematoxylin and eosin (H&E) and Sirius red and scored by experienced pathologists (MP and AH). Steatosis was assessed by the percentage of hepatocytes containing large and medium-sized intracytoplasmic lipid droplets (but not foamy microvesicles), on a scale of 0 to 3 (0, <5%; 1, 5–33%; 2, >33–66%; 3, >66%). Lobular inflammation was scored at 20× magnification ranging from 0 to 3 (0, none; 1, <2 foci per 20× field; 2, 2–4 foci per 20× field; 3, >4 foci per 20× field) when applying the NASH CRN scoring system (18). When applying the Steatosis, Activity, Fibrosis score (SAF score) system, a three-tiered scoring for lobular inflammation was performed ranging from 0 to 2 (0, none; 1, ≤2 foci per 20×; 2, >2 foci per 20×) (19). Hepatocellular ballooning was scored from 0 to 2 (0 = none, 1 = few, 2 = many) and fibrosis scored from 0 to 4 (0 = none, 1 = perisinusoidal or (peri)portal, 2 = perisinusoidal and (peri)portal, 3 = bridging fibrosis, 4 = cirrhosis; not available in one participant). Consequently, the NAFLD Activity Score (NAS) and SAF score could be determined. NAS is the unweighted sum of steatosis grade, lobular inflammation and ballooning and receives a score ranging from 0 to 8. Most cases diagnosed as steatosis have a total NAS score of ≤2, while most cases diagnosed as steatohepatitis have a total score of ≥5. A total score of 3 or 4 can be either steatosis or steatohepatitis. However, NAS has not been developed to establish a diagnosis of NASH, but it is rather a continuous scale of the NASH activity assessment and has thus been used as such in our study (20). Recently, the SAF score has been developed in order to categorise obesity-associated liver disease (21). It assesses the grade of steatosis (S, from S0 to S3), the grade of activity (A from A0 to A4 by the addition of grades of ballooning and lobular inflammation) and the stage of fibrosis (F from F0 to F4). All patients who received a diagnosis of NASH had >5% steatosis in hepatocytes and a grade of activity A ≥ 2 (19, 22).

### Statistical analysis

Data were evaluated for normality of distribution and if necessary, logarithmically transformed. Data are presented as mean ± s.d. for normally distributed data and median (25% percentile–75% percentile) for non-Gaussian distributed data. Kruskal–Wallis tests with Mann–Whitney *U post hoc* tests for continuous variables and the Fisher’s exact test for categorical variables were used for comparison between groups based on the liver parameters (NAS, SAF, grade of steatosis, ballooning, inflammation and fibrosis stage). Spearman correlations were performed to investigate the associations between liver parameters and IR. Groups with less than five participants were excluded from analysis. Furthermore, one-way ANCOVA was conducted to evaluate the differences in HOMA-IR levels while controlling for age, BMI, TG, T2D status, sex and histological components. For ANCOVA the dependent variable was logarithmically transformed. For each ANCOVA output residual plots were made and outliers were removed from the analysis. Test results were considered statistically significant at *P* values <0.05. IBM SPSS statistics (version 25, Chicago, IL, USA) was used for all statistical analysis.

## Results

**Figure 1:**
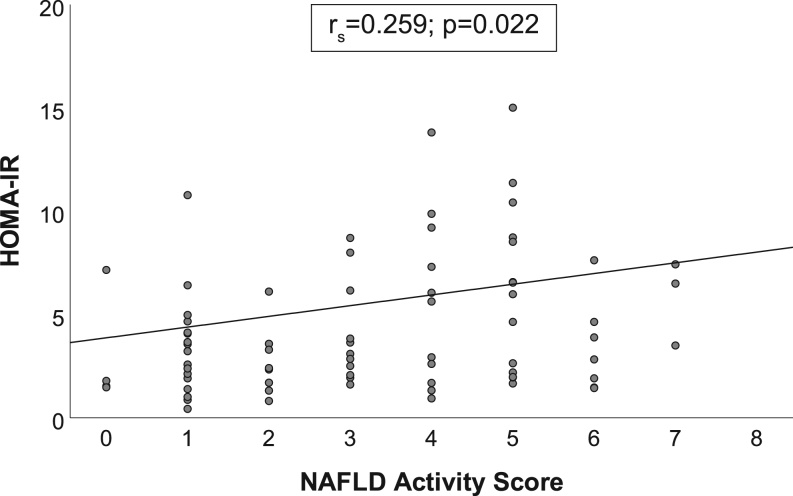
The correlation between NAS and HOMA-IR levels in a population with obesity. HOMA-IR, homeostasis model assessment-estimated insulin resistance.

General characteristics of participants as a whole group and according to SAF-derived NASH status are shown in Table 1. In our cohort, all participants were Caucasian, and 16 subjects (21%) had T2D. Only five participants had no obesity-associated liver disease, 33 had NAFL and 40 NASH according to the SAF score; distribution of participants according to the histopathological components is given in Table 2. Compared to patients with NAFL or without liver disease, patients with NASH showed a trend toward a lower BMI and had higher glucose and TG levels (Table 1). In the whole group, HOMA-IR was positively associated with NAS (Fig. 1), and a trend toward higher HOMA-IR with a more severe SAF classification (*r*_s_ = 0.212, *P* = 0.062) was observed.

**Table 1:** General descriptives of the whole cohort and for SAF groups separately.

	All (*n* = 78)	No NAFLD (*n* = 5)	NAFL (*n* = 33)	NASH (*n* = 40)	*P* value
Age (years)	46 ± 11	42 ± 15	45 ± 12	47 ± 9	0.581
BMI (kg/m^2^)	42.2 ± 4.7	44.0 ± 4.9	43.3 ± 4.4	41.0 ± 4.7	0.073
Sex (male/female)	70/8	3/2	29/4	38/2	0.061
T2D (yes/no)	16/62	0/5	1/32	15/25	**<0.001**
CRP (nmol/L)	40.0 (21.0–61.9)	59.1 (41.9–76.2)	40.0 (25.7–61.9)	37.6 (20.0–57.6)	0.618
Cholesterol (mmol/L)^a^	4.99 ± 1.09	4.50 ± 0.12	4.82 ± 1.03	5.25 ± 1.20	0.431
LDL (mmol/L)^a^	2.86 ± 0.81	2.58 ± 0.29	2.87 ± 0.87	2.89 ± 0.80	0.754
HDL (mmol/L)^a^	1.02 ± 0.33	1.09 ± 0.23	1.01 ± 0.28	1.00 ± 0.41	0.674
TG (mmol/L)^b^	1.84 (1.39–2.42)	1.22 (1.19–1.34)	1.95 (1.53–2.20)	1.97 (1.45–2.89)	**0.030**
NEFA (mEq/L)	0.61 (0.50–0.70)	0.86 (0.79–0.93)	0.564 (0.49–0.65)	0.645 (0.52–0.74)	0.099
Glucose (mmol/L)	5.38 (4.83–6.22)	4.88 (4.66–6.22)	5.11 (4.77–5.55)	5.80 (5.13–6.80)	**0.014**
Insulin (pmol/L)	92.9 (57.1–173.6)	57.1 (49.3–173.6)	92.2 (60.5–152.8)	99.4 (63.2–178.2)	0.472
HOMA-IR	3.40 (1.94–6.50)	1.78 (1.53–4.66)	3.22 (1.91–4.97)	3.76 (2.12–7.53)	0.177

Data represented as mean ± s.d. or median (first quartile–third quartile). *P* values are shown for differences between the three SAF groups, significant *P* values were indicated in bold.

^a^24 patients were excluded from analysis due to use of statins. ^b^One patient is excluded as it had an extreme high value (TG = 16.14 mmol/L).

BMI, body mass index; CRP, C-reactive protein; HDL, high-density lipoprotein; HOMA-IR, homeostasis model assessment-estimated insulin resistance; LDL, low-density lipoprotein; NAFL, nonalcoholic fatty liver; NAFLD, nonalcoholic fatty liver disease; NASH, nonalcoholic steatohepatitis; NEFA, non-esterified fatty acids; T2D, type 2 diabetes; TG, triglycerides.

**Table 2:** Distribution of the population according to the histological NAFLD components.

Histological components NAFLD	*n*
Steatosis	
Steatosis <5%	5
Steatosis 5–33%	31
Steatosis >33–66%	18
Steatosis >66%	24
Ballooning	
No balloon cells	33
Few balloon cells	29
Many balloon cells	16
Lobular inflammation	
No inflammatory foci (per 20× field)	29
<2 inflammatory foci (per 20× field)	42
2–4 inflammatory foci (per 20× field)	6
>4 inflammatory foci (per 20× field)	1
Fibrosis^a^	
No fibrosis	17
Perisinusoidal or (peri)portal fibrosis	39
Perisinusoidal and (peri)portal fibrosis	17
Bridging fibrosis	3
Cirrhosis	1

^a^One patient did not have a fibrosis score.

NAFLD, nonalcoholic fatty liver disease.

**Figure 2:**
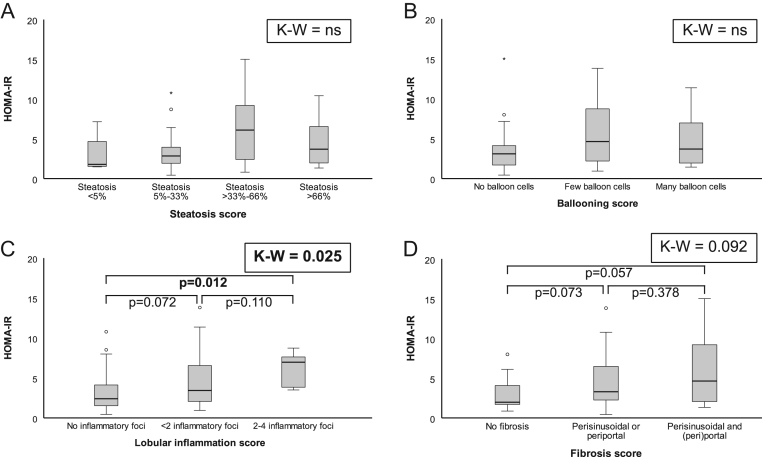
Differences in HOMA-IR levels between histological NAFLD groups. HOMA-IR according to steatosis score (panel A), ballooning score (panel B), lobular inflammation score (panel C) and fibrosis score (panel D). For all panels two data points with a HOMA-IR level above 20 are not shown in the boxplots. In panel C the group with more than four inflammatory foci only consisted of one patient and is excluded from analysis and the graph. In panel D the groups with scores 3 and 4 are excluded from analysis and this figure as those groups consisted of three and one patient, respectively. Statistically significant differences are indicated in bold in the figures. HOMA-IR, homeostasis model assessment-estimated insulin resistance; K-W, Kruskal–Wallis.

There were no differences in HOMA-IR or insulin levels between groups according to grade of steatosis (for HOMA-IR Fig. 2, panel A), whereas higher glucose levels were found in patients with steatosis grade >33–66% compared to those with a lower (*P* = 0.002) or higher (*P* = 0.021) steatosis grade. Similarly, no differences in insulin levels or HOMA-IR were found according to ballooning status, but participants with few ballooning had higher glucose levels compared to those without (Fig. 2, panel B). In contrast, HOMA-IR and insulin, but not glucose levels, were higher in patients with more inflammatory foci compared to those with less (Fig. 2, panel C), and patients with fibrosis score 1 or 2 had higher glucose levels (*P* = 0.006 and *P* = 0.042, respectively) and showed trends towards higher insulin levels and HOMA-IR (Fig. 2, panel D) as compared to those with score 0.

Univariate correlations yielded similar results and are shown in Table 3. Steatosis score was not associated with glucose, insulin or HOMA-IR levels; ballooning score correlated positively with glucose levels only; and lobular inflammation and fibrosis stage showed positive correlations with glucose and insulin levels and with HOMA-IR.

**Table 3:** Correlations between individual histological components and characteristics of glucose metabolism.

	Steatosis (*n* = 78)	Inflammation (*n* = 78)	Ballooning (*n* = 78)	Fibrosis (*n* = 77)
Age (years)	0.096 (0.403)	0.040 (0.726)	0.101 (0.380)	0.097 (0.403)
BMI (kg/m^2^)	−0.053 (0.647)	−0.085 (0.461)	−0.195 (0.086)	0.008 (0.943)
TG (mmol/L)^a^	**0.251 (0.028)**	**0.232 (0.042)**	0.171 (0.137)	**0.242 (0.035)**
NEFA (mEq/L)	−0.144 (0.305)	0.145 (0.299)	−0.071 (0.612)	0.090 (0.527)
Glucose (mmol/L)	0.173 (0.130)	**0.226 (0.046)**	**0.263 (0.020)**	**0.299 (0.008)**
Insulin (pmol/L)	0.170 (0.137)	**0.293 (0.009)**	0.152 (0.183)	**0.280 (0.014)**
HOMA-IR	0.186 (0.102)	**0.327 (0.004)**	0.187 (0.101)	**0.302 (0.008)**

Results are represented as the correlation coefficient (*P* value). Significant *P* values are indicated in bold.

^a^One patient is excluded as it had an extreme high value (TG = 16.14 mmol/L).

BMI, body mass index; TG, triglycerides; NEFA, non-esterified fatty acids; HOMA-IR, homeostasis model assessment-estimated insulin resistance.

Finally, multivariate analyses showed that the difference in HOMA-IR according to grade of lobular inflammation was independent of age and BMI (F(2,72) = 5447; *P* = 0.005); age, BMI and TG (F(2,70) = 5304; *P* = 0.007); age, BMI and sex (F(2,71) = 5436; *P* = 0.006); age, BMI and T2D status (F(2,71) = 3014, *P* = 0.040); and age, BMI and fibrosis score (F(2,65) = 3792; *P* = 0.028). The difference in HOMA-IR levels between fibrosis groups was independent of age and BMI (F(2,68) = 3620; *P* = 0.032); age, BMI and TG (F(2,66) = 3323; *P* = 0.042); and age, BMI and sex (F(2,67) = 3424; *P* = 0.038) but lost its significance when controlling for age, BMI and T2D status (F(2,67) = 2422, *P* = 0.097) and age, BMI and inflammation score (F(2,65) = 1061; *P* = 0.352).

## Discussion

In this population of severely obese subjects, NAFLD severity according to NAS is associated with higher IR. Moreover, from the four histopathological components, lobular inflammation shows the strongest link with IR, whereas grade of fibrosis is no longer related to IR after controlling for inflammation score.

Only few other human studies investigated this topic using histological data. In accordance with our findings, Bril *et al*., who recruited subjects from the general population, found lower whole-body insulin clearance with increasing grade of inflammation (15). Similarly, Ballestri *et al.* and Park *et al.* also report a positive association between IR and lobular inflammation, although this was not found by Jung *et al.* and Petta *et al.* (11, 12, 13, 23). Also in line with our findings, all these studies, except for those by Jung *et al.* (23) and Bril *et al.* (15), report positive associations between indices of IR and fibrosis score (11, 12, 13, 24). However, none evaluated whether fibrosis is associated with IR independently of other NAFLD features, whereas in our cohort, fibrosis was no longer associated with IR when correcting for inflammation. Obviously, NAFLD is a progressive disease and inflammation is considered to be the precursor of fibrosis (25), which might also explain part of the divergence in these findings. Further, contrasting our findings and those of Park *et al.* (in 24 children with NAFLD) (12), other studies showed higher HOMA-IR in patients with a higher grade of steatosis or ballooning (11, 12, 13, 14, 24, 26), whereas Bril *et al*. found that hepatic IR already is present in subjects with simple steatosis and that ballooning, but not steatosis grade, was associated with whole-body IR (15). Differences in methodology and populations could contribute to these discrepant results. We recruited patients who met the criteria for undergoing GBS and willing to undergo a liver biopsy, while other studies recruited subjects already diagnosed with NAFLD. More or less as a result of these different recruitment strategies, our population presented with lesser NAFLD severity, with 33.3% of our population having NASH while in the populations of Petta *et al.* and Jung *et al.* 62.2 and 66.7% have NASH, respectively (13, 23). Further, not all studies corrected for BMI, whereas due to the recruitment strategy our population is more obese and also more insulin resistant (although our participants’ values corroborate with those of similar cohorts (11, 23, 24, 27)), while other studies even included non-obese subjects. Finally, although a recent meta-analysis showed no relation between grade of obesity and NAFLD severity (28), previous reports suggested higher BMI in patients with NASH compared to those with NAFL (11, 12, 13, 14, 23, 24, 26). Subjects with NASH in our study, however, tended to present with lower BMI than participants without NASH which raises the question whether our NASH cohort reflects a subgroup of patients who present with more severe consequences of obesity. Similarly, prevalence of T2DM was highest in those patients with NASH. This could be a confounding factor and when excluding these patients from analysis the positive association between HOMA-IR and NAS lost significance (data not shown). However, as diagnosis of T2D is based on arbitrary cut-offs, while IR is a physiological condition and all patients stopped intake of antidiabetic drugs timely before sampling; this will not have affected our main findings. In summary, our study is the first to address the relation between IR and components of NAFLD in a homogenous adult population with severe obesity (BMI >35 kg/m^2^) and suggests that in this specific population hepatic inflammation is the component most strongly associated with IR.

Several pathophysiological processes might explain these findings. In obesity-related IR, diminished inhibition of hormone-sensitive lipase will lead to enhanced efflux of FFA from adipocytes, while the compensatory systemic hyperinsulinaemia increases hepatic FFA uptake and activates lipogenic genes (29, 30, 31). Once excessive influx of FFA overflows the hepatocytes’ capacity to store them as TG in lipid droplets, accumulation of diacylglycerols (DAGs) will impair insulin signalling causing decreases in hepatic glycogen synthesis and increases in gluconeogenesis (6, 8, 30, 32, 33, 34, 35, 36). As histological grading of hepatic steatosis is based on the amount of lipid droplets and not intracellular DAG content (which we were unable to reassess in our samples), this could explain the absent association between grade of steatosis and IR in our cohort. Further, as DAG accumulation also leads to production of reactive oxygen species causing hepatic inflammation (37, 38), lobular inflammation seems a consequence rather than a cause of obesity-related IR. A view which is also supported by some animal data (39). However, there is also evidence suggesting a contribution of hepatic inflammation to systemic IR. For instance, in hepatitis C virus infection, hepatic inflammation increases circulating levels of interleukin 1, tumour necrosis factor alpha and interleukin 6, which all can stimulate IR (40, 41). In addition, other processes such as alterations in hepatokine secretion or ceramide metabolism probably also contribute to the association between hepatic inflammation and IR (42, 43).

Although our study is limited by its cross-sectional design and the lack of a blinded and single histopathological evaluation, our findings are of clinical importance (44). For instance, clinical NAFLD evaluation still relies in large part on imaging procedures that focus on hepatic fat content and to a lesser extent on fibrosis whereas grade of inflammation is more difficult to assess. However, in contrast to fibrosis, inflammation is considered to be a reversible state of NAFLD so accurate estimation of this component of NAFLD is of clinical relevance. Moreover, from our findings it follows that obese subjects with NAFL who proceed to NASH should be screened for IR, and that, in addition to screening for NAFLD in patients with T2D, obese patients with uncomplicated IR should also be evaluated for NALFD (44, 45).

In conclusion, in subjects with severe obesity, IR is most strongly associated with lobular inflammation, more than with other histopathologic components of NAFLD. This may reflect a direct relation between hepatic inflammation and systemic insulin signalling but could also be explained by a subgroup of patients which is more prone to develop obesity-related consequences.

## Declaration of interest

The authors declare that there is no conflict of interest that could be perceived as prejudicing the impartiality of the research reported.

## Funding

The SMELSS was supported by a grant from the Fund for Scientific Research – Flanders (FWO-Vlaanderen, grant 1517316N).

## Author contribution statement

Y Van Nieuwenhove and B Lapauw contributed equally to this work.
